# Thoracolumbar spinal cord injury: management, techniques, timing

**DOI:** 10.1007/s00068-024-02595-8

**Published:** 2024-07-17

**Authors:** Marko Jug, Radko Komadina, Klaus Wendt, Hans Christoph Pape, Frank Bloemers, Christoph Nau

**Affiliations:** 1 University Medical Centre Ljubljana, Medical Faculty, University of Ljubljana, Ljubljana, Slovenia; 2 Medical Faculty, University of Ljubljana, Ljubljana, Slovenia; 3 University Medical Center Groningen, University of Groningen, Groningen, The Netherlands; 4 University Hospital of Zürich, University of Zürich, Zürich, Switzerland; 5 Amsterdam University Medical Centre, Vrije Universiteit Amsterdam, Amsterdam, The Netherlands; 6 University Hospital Frankfurt, Goethe University, Frankfurt, Germany

**Keywords:** Spinal cord injury, Thoracic spine, Thoracolumbar spine, Management, Timing, Decompression

## Abstract

Acute traumatic spinal cord injury (tSCI) is a complex and heterogeneous injury, where the level of injury, injury severity, duration and degree of spinal cord compression, and blood pressure management seem to influence neurologic outcome. Although data in the literature seem to be inconsistent regarding the effectiveness of surgical decompression and spinal fixation in patients with thoracic and thoracolumbar tSCI, some single-center studies suggest that early surgical decompression may lead to a superior neurologic outcome, especially in patients with incomplete tSCI, suggesting surgical decompression to be performed as soon as possible. However, high energy injuries, especially to the upper thoracic levels, may be too severe to be influenced by surgical decompression, which may represent a critical second hit for the polytraumatized patient. Therefore, the surgeon first needs to critically evaluate the potential for neurologic recovery in each patient before determining the ideal timing of surgery. Circulatory stabilization must be achieved before surgical intervention, and minimally invasive procedures should be preferred. Invasive blood pressure monitoring should be started on admission, and maintenance of a MAP between 85 and 90 mmHg is recommended for a duration of 5–7 days, with special attention to the prevention of hypoxia, fever, acidosis and deep venous thrombosis. The role of a 24-hour infusion of high-dose MPSS is still controversial, but it may be offered at the discretion of the treating surgeon to adult patients within 8 h of acute tSCI as a treatment option, especially in the case of very early decompression or incomplete tSCI.

## Introduction

Acute traumatic spinal cord injury (tSCI) is a complex and heterogeneous injury, where the level of injury, injury severity, duration and degree of spinal cord compression, and blood pressure management seem to influence neurologic outcome [1–4]. Recent guidelines suggest early surgical decompression of the injured spinal cord within 24 h of injury regardless of the level of injury [5]. However, although there is growing evidence supporting early decompression in cervical trauma [4, 6–8], there is still controversy regarding the ideal timing for decompression in the setting of thoracic and thoracolumbar SCI [9]. Nevertheless, the neurologic outcome after tSCI depends on the primary and secondary injury, and mitigating secondary injury represents a key target for intervention in the acute phase [1]. In this regard, early decompressive surgery, arterial blood pressure augmentation and methylprednisolone sodium succinate (MPSS) administration have been suggested as treatment options in the acute phase [1].

## Who can benefit from surgery?

As tSCI represents a heterogeneous group of injuries, and TL spinal injuries with tSCI are frequently found in polytraumatized patients [10, 11], who are susceptible to second hit injury by unnecessary surgery, one of the most important questions to be answered first is who can benefit from surgery, especially early surgery.

In the case of TL spinal fractures, surgery is performed for spinal realignment and stabilization. In addition, spinal stabilization within 48 to 72 h, especially of the thoracic spine, has a beneficial effect on pulmonary function, and the outcome of polytrauma patients with lumbar and thoracic spinal injuries seems to be superior upon early spinal surgery within 48 to 72 h [10, 11]; however, the effect of very early surgical spinal cord decompression in patients with tSCI on neurologic recovery remains unclear. Especially in the hemodynamically unstable patient an urgent surgical decompression may have detrimental effects. From this point of view, the surgeon should be able to first evaluate the probability of the effect of surgical decompression on neurologic recovery and on the patient’s general condition, as thoraco-lumbar spinal decompression usually represent major operations. Although stabilization of the spine can be achieved with new less invasive percutaneous techniques, the surgeon should be able to predict the effect of decompression before putting the patient at irrational risk.

A recent study evaluated not only the effect of timing of surgical decompression on neurologic recovery after acute tSCI in patients with cervical spinal fractures, but also other factors that might influence neurologic outcome. The results suggest that although shortening the time to surgical decompression has a significant influence on neurologic outcome, the severity of tSCI, in other words, complete or incomplete injury, and the degree of spinal canal compromise, in other words, the degree of compression of the injured spinal cord, at admission play an important role as well. It was reported that patients with more than 60% of spinal canal compromise and a complete tSCI at admission did not experience any significant neurologic recovery, even in the case of surgical decompression in the first hours after injury, suggesting that some patients with very severe tSCI and spinal dislocation may not benefit from early surgery at all [2].

Although the study evaluated patients with cervical injuries, the results provide an insight into other factors that may determine neurologic outcome after tSCI. This insight may be very important especially in respect of decision-making in polytraumatized patients with thoracic injuries. Patients with complete thoracic tSCI have a reduced potential for neurologic recovery compared to patients with complete cervical tSCI [4]. A trend towards poorer outcome was reported in patients with higher thoracic tSCI in comparison to lower thoracic and thoracolumbar tSCI [12]. A higher energy injury mechanism [13], scarcer blood supply of the spinal cord [14], and a narrower spinal canal are believed to play a role in greater tissue disruption in the thoracic region. On the other hand, the potential for neurologic recovery in incomplete thoracic, thoracolumbar and cervical tSCI seems to be similar [4].

**Fig. 1:**
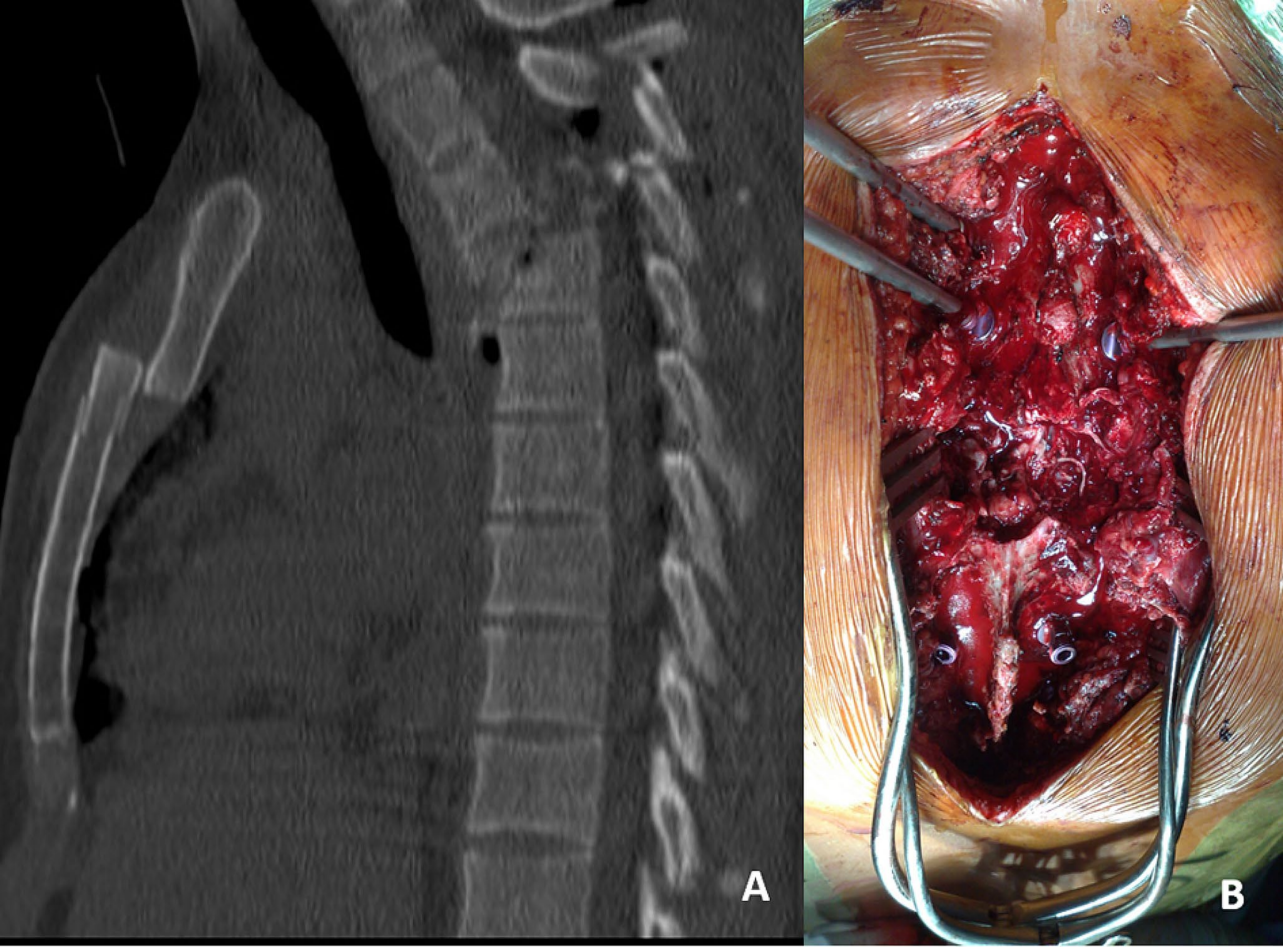
**a** A case of a polytraumatized patient with complete tSCI and thoracic spine injury (fracture-dislocation at T3 level, sternum fracture, ribs and both scapular fracture, spleen rupture) after a gliding accident. The patient first underwent splenectomy and was subjected to vigorous resuscitation in the ICU for 24 h. The next day operative spinal reduction and stabilization was performed. **b** During the operation a torn spinal cord was found at the level of spinal injury. Posterior reduction and spinal fixation with dural reconstruction was performed. The patient had no neurologic recovery

**Fig. 2:**
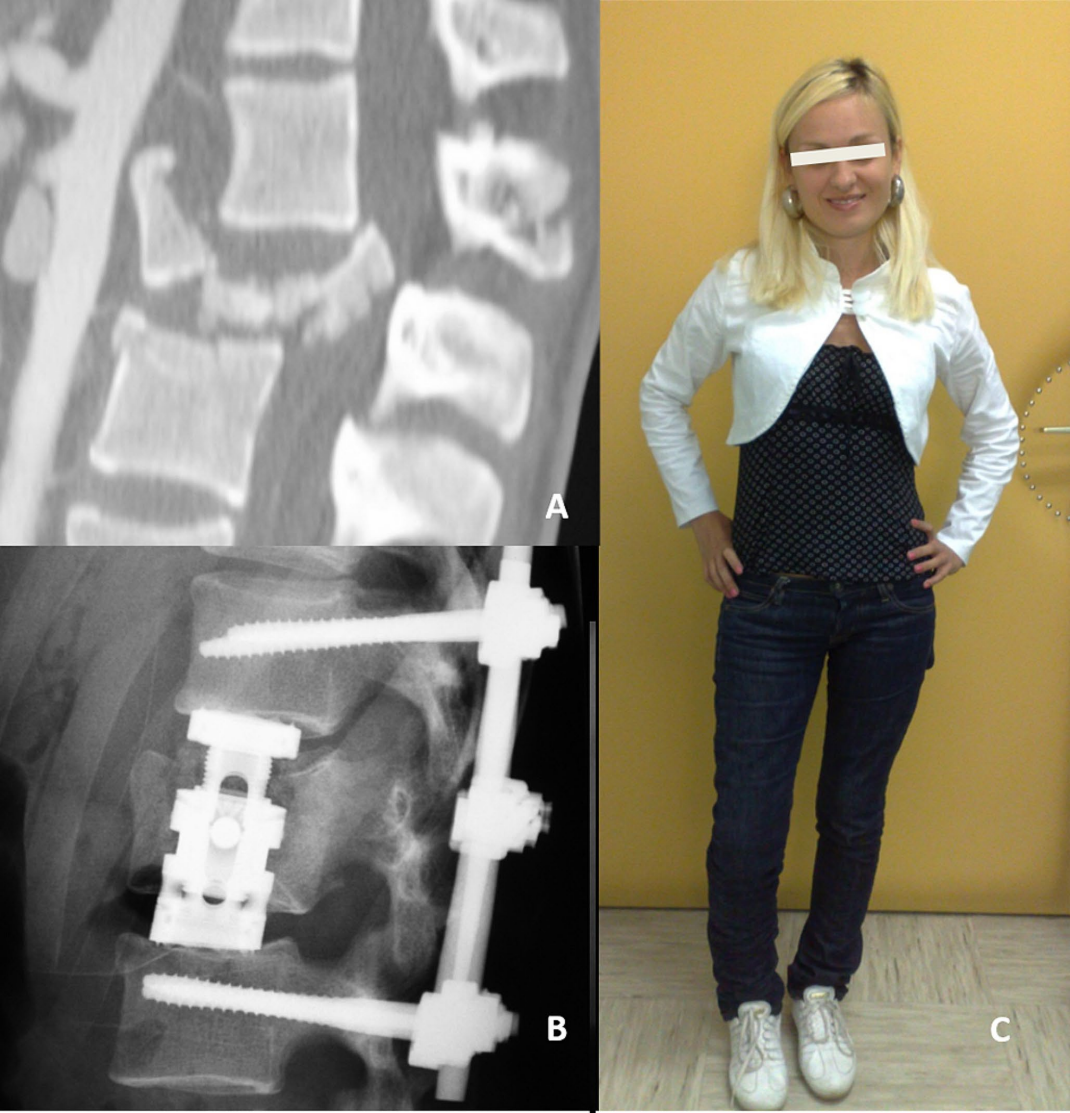
**a** A case of a polytraumatized patient with complete tSCI and multiple injuries (fracture-dislocation at L1 level, pelvis fracture, both iliac arteries dissection, long bones injuries, etc.) after a suicide jump injury. **b** The patient first underwent arterial embolisation and vigorous resuscitation in the ICU for 12 h; immediately after circulatory stabilization, a posterior spinal stabilization and realignment was performed with indirect spinal cord decompression (validated by intraoperative CT); two weeks after injury an additional anterior approach was performed for anterior column reconstruction. **c** One year after injury the patient experienced a complete neurologic recovery

These data suggest that in the case of complete tSCI and prominent dislocation in the high thoracic region the goal of surgery is predominantly the stabilization of the thorax and realignment and stabilization of the spine, whereas spinal decompression for tSCI seems of secondary importance (Fig. 1). On the other hand, in the case of lower thoracic and thoraco-lumbar regions (Fig. 2), or especially in spinal injuries under the level of the spinal cord, where nerve roots may resist even major obliterations of the spinal canal, or in the case of incomplete tSCI, early spinal decompression seems to be more effective in regard to neurologic recovery and should be performed as soon as possible.

## Timing of decompressive surgery and haemodynamics

Although guidelines suggest early surgical decompression of the injured spinal cord within 24 h of injury regardless of the level of injury [5], a recent multicenter prospective cohort study that compared early surgical decompression following acute tSCI (within 12 h of injury) to late surgical decompression (12 to 72 h after injury) did not show statistically significant or clinically meaningful neurologic improvements 12 months after injury [15]. However, this study recruited patients from 17 centers in Europe and compared heterogeneous tSCI with different spinal levels of injury, which in our view represents a serious study limitation. On the other hand, there are growing data supporting early decompression in cervical trauma [4, 6–8], whereas the effect of early surgical decompression regarding thoracic and thoracolumbar SCI is still controversial, which emphasizes the potential difference in outcome between different levels of spinal injury [9]. In line with this observation, a study group from the Nederlands did not observe a significant beneficial effect of surgical decompression within 24 h of injury in patients with thoracic and thoracolumbar tSCI in a meta-analysis [12]. Conversely, a recent randomized control trial showed that surgical decompression within 24 h of acute traumatic thoracic and thoracolumbar SCI is safe and associated with improved neurologic outcomes [16]. Moreover, a better functional outcome was reported in patients who underwent surgical decompression within 8 h of injury compared to later decompression in both the thoracic and the thoracolumbar spine [17, 18]. However, the positive effect of early surgery on neurologic recovery is more evident in incomplete tSCI [9, 19], as in patients with complete injuries the primary injury may be so severe that no intervention can result in neurologic improvement [9]. These observations are in line with previous studies that reported superior neurologic recovery in patients with cervical tSCI who underwent surgical decompression within the first 8 h of injury compared to later time windows [2, 7, 8], and patients with incomplete injuries [2, 7]. Taken together, these data support the “TIME IS SPINE” concept, which emphasizes the biological rationale for decompressive surgery as soon as possible after tSCI in order to mitigate secondary injury [20]. This calls into question the suggested time window of 24 h as the ideal time for decompression, emphasizing the importance in avoiding the comparison of a too heterogeneous cohort.

Accordingly, posterior reduction, decompression and fixation of thoracic and thoracolumbar spinal injuries is suggested to be performed as damage control surgery aimed to enhance spinal cord perfusion as soon as possible after injury, and should not be postponed for nonmedical reasons, especially in patients with incomplete injuries. Minimally invasive procedures with intraoperative CT validation of successful decompression may be preferred to shorten the time of operation, reduce blood loss and intraoperative hypotension, which may result in a second hit to the patient and the spinal cord itself. Namely, spinal cord compression from bone fragments, hematoma and dura increases the intraspinal pressure (ISP), resulting in a drop in spinal cord perfusion pressure (SCPP), which correlates with a poor neurologic outcome after tSCI [21, 22]. However, SCPP not only depends on ISP but also on mean arterial blood pressure (MAP) [21]. Immediate invasive MAP monitoring and management is therefore suggested to prevent hypotension, as tSCI is often additionally complicated by neurogenic shock and/or polytrauma [23]. A whole-body CTA is suggested to rule out any concomitant critical injuries, and circulatory stabilization is mandatory before surgical decompression. Recent guidelines suggest a target MAP between 85 and 90 mmHg for at least 5–7 days after injury [3], although the optimal MAP likely varies between patients depending on their ISP [22, 24, 25]. In fact, bony decompression alone may fail to prevent the rise in ISP second to spinal cord edema and the limited compliance of the dural sac, resulting in an intradural compartment syndrome despite laminectomy and bony decompression [26]. Expansion duroplasty has been suggested for intradural space expansion and ISP lowering [26]. Additional data regarding the effectiveness and morbidity of duroplasty are needed before recommendations can be made. On the other hand, ISP monitoring by an intradural probe may represent a minimally invasive but valuable tool, and may be considered in the first week after injury for SCPP optimization in the intensive care unit [27]. In addition, management of hypoxia, fever, and acidosis is suggested to improve local spinal cord metabolism [22], and prophylaxis to prevent deep venous thrombosis should be administered as soon as possible [1]. It has been shown that very early surgical decompression is feasible only in patients who are transferred directly from the site of injury to a specialized center [7]; therefore, a direct transfer of all tSCI patients from the site of injury to a hospital capable of definitive care is recommended.

## Methylprednisolone sodium succinate

The use of methylprednisolone sodium succinate (MPSS) in tSCI has been a matter of dispute since its introduction, and recommendations evolved from an option in the treatment of acute tSCI in the 2002 AANS/CNS guideline to recommendation against the use of MPSS in the 2013 updated version [13], with no compelling evidence-based rationale to justify the substantial change. Therefore, the 2017 AOSpine guideline aimed to bridge the gap between the 2002 and 2013 AANS/CNS guidelines, suggesting a 24-hour infusion of high-dose MPSS be offered to adult patients who present within 8 h of acute tSCI as a treatment option regardless of the level of injury [28]. Although a recent meta-analysis argues against the use of high-dose MPSS treatment in the context of acute SCI on the one hand [29], it is important to note that available evidence is based on studies that did not evaluate the effect of MPSS therapy in patients who underwent very early decompression and SCPP optimization. In fact, failure to optimize SCPP impairs drug delivery at the injury site, which may lead to the erroneous conclusion that the tested drug does not improve outcome after tSCI [24]. Therefore, a 24-hour infusion of high-dose MPSS may be offered to adult patients within 8 h of acute SCI as a treatment option, especially patients who undergo very early decompression or present with incomplete tSCI.

## Conclusions

A direct transfer of all tSCI patients from the scene to a hospital capable of definitive care is recommended. The potential for neurologic recovery after surgical decompression has to be evaluated and encompassed in the decision-making process regarding the timing of surgery. However, a posterior reduction, decompression and fixation of thoracic and thoracolumbar spinal injuries is recommended as soon as possible after circulatory stabilization, especially in patients with incomplete injuries. Invasive blood pressure monitoring and maintenance of a MAP between 85 and 90 mmHg is recommended for a duration of 5–7 days. Management of hypoxia, fever, acidosis and deep venous thrombosis prophylaxis is also recommended. A 24-hour infusion of high-dose MPSS may be offered to adult patients within 8 h of acute tSCI as a treatment option, especially in the case of very early decompression or incomplete injury.

## Data Availability

No datasets were generated or analysed during the current study.
